# Effect of *Cyberlindnera jadinii* yeast as a protein source on intestinal microbiota and butyrate levels in post-weaning piglets

**DOI:** 10.1186/s42523-020-00031-x

**Published:** 2020-05-05

**Authors:** Stanislav Iakhno, Özgün C. O. Umu, Ingrid M. Håkenåsen, Caroline P. Åkesson, Liv T. Mydland, Charles McL. Press, Henning Sørum, Margareth Øverland

**Affiliations:** 1 Department of Food Safety and Infection Biology, Faculty of Veterinary Medicine, Norwegian University of Life Sciences, Oslo, Norway; 2 Department of Animal and Aquacultural Sciences, Faculty of Biosciences, Norwegian University of Life Sciences, Ås, Norway; 3 Department of Basic Sciences and Aquatic Medicine, Faculty of Veterinary Medicine, Norwegian University of Life Sciences, Oslo, Norway

**Keywords:** Pig microbiota, Yeast diet, *Cyberlindnera jadinii*, Gut, Butyrate, Crypt depth, 16S *rRNA* gene sequencing, *Lactobacillus* spp.

## Abstract

**Background:**

Dietary yeast inclusions in a pig diet may drive changes both in gut bacterial composition and bacterial functional profile. This study investigated the effect of *Cyberlindnera jadinii* as a protein to replace 40% of the conventional proteins in a diet for weanling pigs on the microbiota in the small and large intestine, colonic short-chain fatty acid concentration, and colonic histopathology parameters. Seventy-two pigs weaned at 28 days of age were randomly assigned to either a control or a *C. jadinii*-based diet and followed for 2 weeks.

**Results:**

Compared with the controls, higher numbers of cultivable lactic acid-producing bacteria in the small and large intestine were registered in the yeast group. Alpha and beta bacterial diversity were different between the diet groups with lower alpha-diversity and distinct bacterial composition in the large intestine in the yeast group compared with those of the controls. The large intestine microbiota in the yeast group had higher numbers of *Prevotella*, *Mitsuokella* and *Selenomonas* compared with those of the controls. The concentrations of colonic acetate and butyrate were higher in the controls compared with that of the yeast group. The colonic crypt depth was deeper in the control group. The gut histopathology of colonic tissues revealed no differences between the diets.

The colonic crypt depth tended to be deeper with higher relative abundance of an unclassified Spirochetes, higher colonic butyrate concentration, and higher bacterial richness. The concentration of colonic butyrate was positively associated with the relative abundance of the *Faecalibacterium prausnitzii*, *Dialister*, and an unclassified amplicon of the Spirochaetaceae family in the colon.

**Conclusions:**

The replacement of the conventional proteins by proteins from *Cyberlindnera jadinii* in a weanling pig diet reshaped the large intestine microbiota structure. The novel yeast diet appeared to be selective for *Lactobacillus* spp., which may represent an added value resulting from using the sustainably produced yeast protein ingredient as an alternative to conventional protein ingredients in animal diets. The large intestine bacterial composition and their metabolites may be involved in an adaptive alteration of the colonic crypts without pathological consequences.

## Introduction

Up to 70% of globally produced soy is used to maintain livestock production [1]. Sustainable protein alternatives are needed to reduce the dependency on soy and other conventional proteins as ingredients in the feed for animal husbandry. As early as in the 1940’s, researchers pursued the idea of replacing a substantial fraction of protein in animal feed with proteins from yeast derivatives [2, 3]. It soon became apparent that the lack of cost efficient methods for large scale yeast production would limit the use of yeast proteins [3]. Moreover, additional costs arose from the necessity of vitamin D and calcium supplementation to counteract the rachitogenic effect of yeast diets [4, 5]. Today, technology exists to produce yeast by industrial fermentation of *Picea abies* second-generation sugars as a carbon and energy source [6]. Our previous work has shown that up to 40% of conventional protein in a pig diet can be successfully replaced by proteins from a strain *Cyberlindnera jadinii* yeast [7]. The addition of yeast as a protein source supported high growth performance and improved gut health of the weanling pigs [7]. To date, a number of studies have investigated the effect of yeast supplementation on pig microbiota composition. Addition of live *Saccharomyces cerevisiae* to a diet promoted overgrowth of *Mitsuokella* bacterial genus in the large intestine microbiota in weanling piglets [8]. Inclusion of cider yeast probiotic to a diet shifted faecal microbiota towards higher numbers of *Selenomonas* and *Prevotella* in weanling piglets [9]. In addition, Upadrasta and co-workers reported reduction in *Faecalibacterium*, *Roseburia*, and *Eubacterium* in faeces of the yeast group. Impact of yeast derived components such as cell wall β-glucans and mannan-oligosaccharides on the gut microbiota in pigs has also been studied. Fouhse and co-workers reported high relative abundance of *Mitsuokella* and low relative abundance of *Coprococcus* and *Roseburia* in caecum of piglets supplemented with yeast derived mannan-rich fraction [10]. Nakashimada et al. studied changes in pig faecal bacterial composition using an in vitro intestinal model. These investigators found lower numbers of *Faecalibacterium* in the reactor system with addition of yeast cell wall components than without [11]. While supplementation of yeast ingredients does seem to promote distinct intestinal bacterial groups, the reduction in short-chain fatty acid (SCFA) producing bacteria may be another intrinsic feature of such diets. One of the major SCFAs produced by intestinal bacteria, butyrate, is an exogenous metabolite with a number of key functions related to gut homeostasis (reviewed in [12, 13]). While serving as fuel for colonocytes [14, 15], it is debatable whether high molarities of butyric acid are beneficial (reviewed in [16]). For instance, high colonic butyrate concentration is believed to modulate colonic crypt architecture [17], induce apoptosis in the stem cell compartment of crypts [18], and supress crypt stem cell proliferation [19]. Recent publications have been primarily focused on yeast as a feed additive and have investigated the effects of low levels of inclusion of yeast and its components on the gut microbiota in pigs [8, 9, 20]. However, little is known how inclusion of high levels of yeast affects microbial community of intestines. We hypothesize that the novel yeast diet can reshape intestinal microbiota composition in weanling piglets. The reason for featuring the post-weaning period in this study was because of the stress the animals experience during that period [21, 22], which may define the course of animal health development.

We used 16S *rRNA* bacterial gene sequencing and cultivation methods to compare the gut microbial consortia of yeast fed weanling piglets with that of the controls. Also, we investigated a possible role of individual bacterial groups in relation to the large intestine butyrate production and utilization.

## Methods

### Animals, housing, diet allocation

**Fig. 1:**
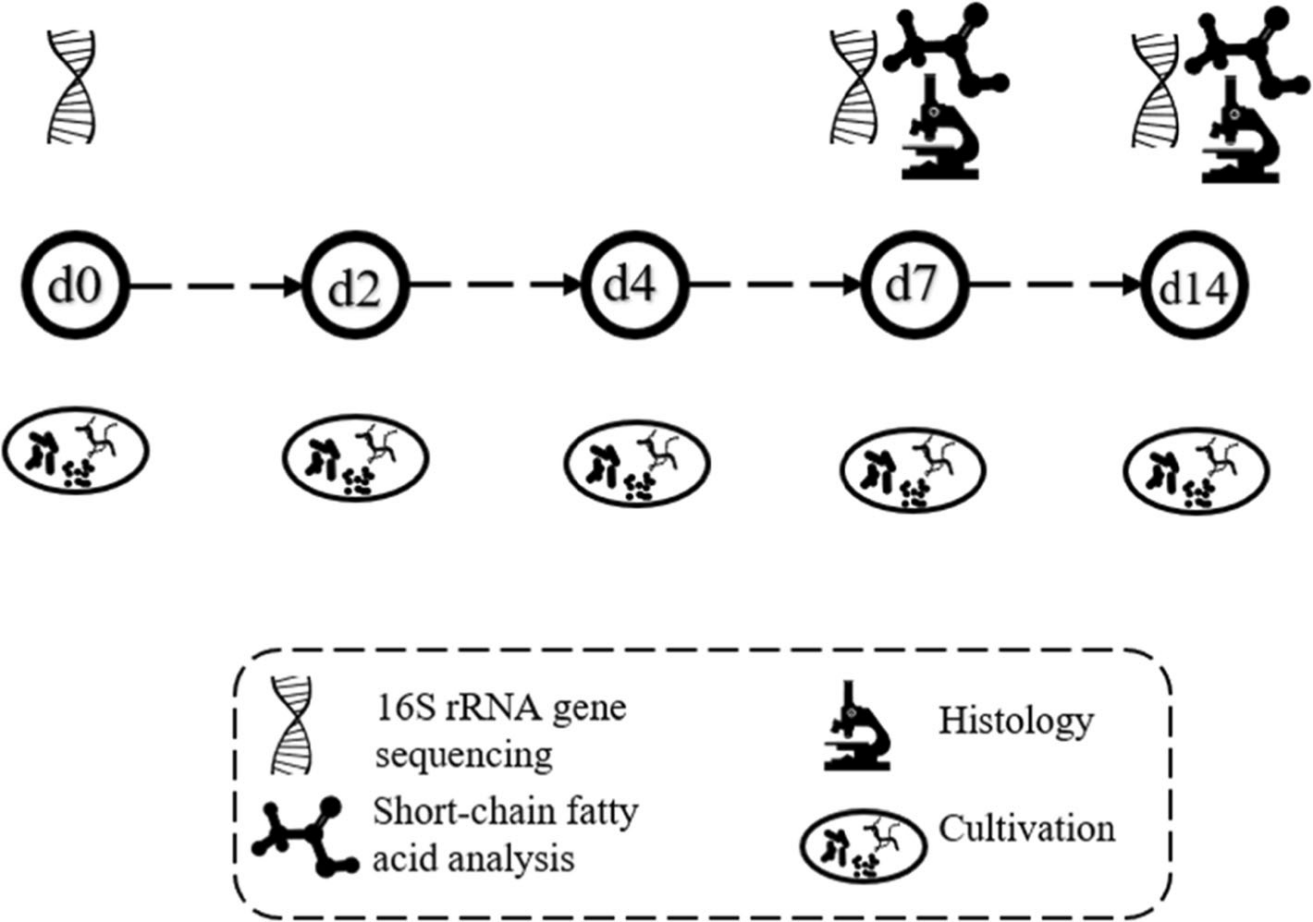
The outline of the study sampling design. The timeline is shown as circles connected by the dashed line from left to right. It starts from day 0 PW (d0 corresponds to 28th postnatal day) and continues till day 14 PW (d14). The sampling procedures are given above (16S *rRNA* gene sequencing, histology, and short-chain fatty acid analysis) and below (cultivation) the timeline

The trial was conducted at an experimental farm of the Norwegian University of Life Sciences (NMBU), Ås, Norway in the fall of 2017. A total of 72 crossbred [(Norwegian Landrace x Yorkshire z-line) x (Duroc) and (Norwegian Landrace) × (Duroc)] weanling piglets, selected from ten litters, was included in the experiment. The piglets were selected to enter the study based on their weight at the day of weaning, and after blocking by litter and body weight, the pigs were randomly allocated to either the control or the yeast diet. All animals were healthy during the nursery period and throughout the experiment. The animals were housed in environmentally controlled pens with a slatted floor at front and roofed resting area with a rubber mat. The animals were introduced to creep feed 2 weeks prior to weaning. The experiment was initiated when the piglets were weaned at 28 days of age (day 0 PW). Five to six piglets were grouped together in each pen and group-fed one of the allocated diets. All animals had ad libitum feeding and access to drinking water throughout the experiment. Diets were formulated to be isonitrogenous and isoenergetic based on the chemical composition of the ingredients and to meet, or exceed, the nutrient requirements of weanling pigs (Table 1). In the yeast diet, 40% of the crude protein derived from *Cyberlindnera jadinii* cells (LYCC 7549; Lallemand Yeast Culture Collection). The yeast cells were processed as described previously [6]. Briefly, after fermentation, the cells were washed, centrifuged, heat-inactivated, and dried. The diets were cereal-based (wheat, barley and oats), and the main protein ingredients in the control diet (soybean meal, potato protein concentrate, fish meal, and rapeseed meal) were partly replaced by yeast meal in the yeast diet (Table 1). At the days 2, 4, 7, and 14 PW, eight animals from each of the two feeding groups were sacrificed followed by sampling (Fig. 1). In addition, eight littermates were sampled at day zero to provide a baseline point for the day of weaning.

**Table 1:** Ingredients (g/kg as fed) and analysed chemical composition (g/kg DM, unless otherwise stated) of experimental diets

	Control diet	Yeast diet
Ingredients, g/kg as fed		
Wheat	627	594
Barley	100	100
Oats	50	50
Soybean meal	80	19
Potato protein concentrate	34	9
Fish meal	20	5
Rapeseed meal	20	5
Yeast - *Cyberlindnera jadinii*	–	146
Rapeseed oil	20	23
Minerals, vitamins and amino acids	49	49
Nutrients, g/kg of DM		
DM, g/kg	869	885
Crude protein	202	194
NDF	110	102
Starch	508	494
Crude fat	45.3	46.2
Ash	52.7	51.2
Phosphorus	8.01	9.08
Gross energy, MJ/kg	18.94	18.96

### Bacterial cultivation / DNA extraction / 16S *rRNA* gene amplicon sequencing

Luminal contents from *ileum distalis*, *apex ceci*, and *apex coli spiralis* were collected. Serial dilutions of 0.1 mg/ml of digesta in 0.9% saline were inoculated onto media. MacConkey, Tryptose Sulfite Cycloserine (TSC), de Man, Rogosa and Sharpe (MRS), and Slanetz and Bartley agar (Oxoid, Cambridge, UK) were used to recover and quantify coliforms, *Clostridium perfringens*, lactic acid bacteria (LAB), and enterococci, respectively. The dilution and incubation schemes were applied as described previously [23].

For 16S *rRNA* gene sequencing, digesta samples from *ileum distalis*, *apex ceci*, and *apex coli spiralis* were snap-frozen in liquid nitrogen and stored at -80 °C until DNA extraction. The DNA extraction was carried out on samples collected on days 0, 7 and 14 PW, according to a previously described protocol [24] with minor modifications. Briefly, 200 mg of thawed gut contents were added to 1 ml of InhibitEX Buffer (QIAGEN, GmbH, Hilden, Germany) following loading 500 mg of zirconia/silica beads (∅ *=* 0.1 mm, Carl Roth, Karlsruhe, Germany). The TissueLyser adaptors were cooled down at − 20 °C for 15 min prior to the bead-beating step. The bead-beating lasted for 1.5 min at 30 Hz in TissueLyser II (Qiagen, Retsch GmbH, Hannover, Germany). Proteins were digested with 30 μL of Proteinase K II (QIAGEN, GmbH, Hilden, Germany). DNA was bound to QIAamp spin column followed by washing with AW1 and AW2 buffers (QIAGEN, GmbH, Hilden, Germany). DNA was eluted with ATE buffer (QIAGEN, GmbH, Hilden, Germany). The yielded DNA purity was assessed by NanoDrop instrument (Thermo Fisher Scientific, Waltham, MA) with subsequent quantification by Qubit fluorometric broad range assay (Invitrogen, Eugene, OR, USA).

The library preparation and amplicon sequencing of V1-V3 hypervariable region of bacterial 16S *rRNA* gene were performed at GATC Biotech AG (Konstanz, Germany) using 27F (5′-AGAGTTTGATCCTGGCTCAG-3′) and 534R (5′-ATTACCGCGGCTGCTGG-3′) primers. A microbiome standard, 20 Strain Staggered Mix Genomic Material (ATCC® MSA-1003™), was used as a positive control. The amplicon sequencing was run in three batches on an Illumina HiSeq 4000 sequencer. The resulting sequences were deposited in the SRA (PRJNA580284).

### Illumina 16S *rRNA* gene amplicon data curation

Forward Illumina demultiplexed reads were taken to the analysis. The reads were analysed using DADA2 R package, version 1.8.0 [25]. The core DADA2 algorithm applied had the following setup: A) Quality filtering parameters: maxEE = 1, truncQ = 2 with forward primer clipping; B) Dereplication and denoising of the quality controlled reads; C) Resulting feature tables obtained from separate Illumina runs were merged with subsequent chimaera removal; D) Taxonomic assignment using RDP Naive Bayesian Classifier implemented in DADA2 R package (default settings). The GreenGenes database, version 13.8, [26] was used as a reference database for taxonomy assignment. The LULU post-clustering algorithm was applied to optimize diversity metrics [27].

### Colonic SCFA, growth performance and liver index measurement

The colon digesta samples at day 7 PW and day 14 PW were the replicate samples of those used for the 16S *rRNA* gene sequencing analysis. The samples were thawed on ice. A mixture of 500 mg of gut contents sample and 500 μl of ice cold ddH_2_O was sonicated for 5 min in cold water. Next, after mixing and centrifugation (15 min, 4 °C, 15000 g), the supernatant was transferred to a spin column (45 kDa). After another centrifugation step (15 min, 4 °C, 15000 g), the samples were spiked with internal standards. Short-chain fatty acids (SCFA) were measured by “TRACE 1300 Gas Chromatograph” with autosampler, “AS 1310” (Thermo Fischer Scientific, Milan, Italy). Parameters of the capillary column were as following: model – “Stabilwax – DA”; length – 30 m; inner diameter – 0.25 mm; film thickness – 0.25 μm (Restek corporation, Bellefonte, PA, USA). The column operating protocol was as follows: starting temperature – 90 °C (2 min); temperature increase - 10 °C/min until 150 °C, 50 °C/min until 250 °C (1 min). The rate of Helium flow was 3 mL/min. Concentrations of acetic, propionic acids, as well as butyric, valeric acids and their isomers, were reported in μmol per gram of intestinal contents. The average daily gain (ADG) in this study was calculated as: (slaughter day body weight – body weight at weaning) / number of days PW. The liver index was calculated as used previously in [7]: liver index = liver weight (kg)/ live body weight (kg).

### Histology

Colon tissue samples were collected within 20 min of euthanasia and fixed in 10% formalin. The gut contents were emptied, and mucosal surface was rinsed gently with cold water prior to formalin fixation. After 48 h of fixation, the tissues were routinely processed, embedded in paraffin and 4 μm sections were mounted on glass slides. The sections were subsequently deparaffinized in xylene and rehydrated in graded alcohol before routine staining with haematoxylin and eosin. The colonic tissues were evaluated histopathologically and scored semi-quantitatively where no pathology was scored 0, very mild tissue changes received the score 1, mild changes 2, moderate changes 3, and severe changes 4. Formalin-fixed, paraffin-embedded tissue sections were also stained with high iron diamine and alcian blue (HID-AB). Digital images of the intestinal sections were captured using NanoZoomer (Hamamatsu Photonics). Morphometric measurements were performed using the software Aperio Image Scope v12.3.3.5048 (Copyright Leica Biosystems Pathology Imaging, 2003–2016). For crypt depth (CD) measurements, the ten longest and well oriented crypts were selected, and micrographs were captured at 10× magnification. CD was measured from the crypt opening at the mucosal surface to the deepest portion of the crypt adjacent to the *tunica muscularis mucosae*.

### Statistical analysis

The sample-size estimation was based on our pilot study with the similar design and feed composition (unpublished). To compare bacterial average CFUs recovered from selective plates, the non-parametric Mann-Whitney-Wilcoxon (MWW) test was applied. The linear regression model was used to predict variance in LAB colony-forming unit (CFU) with the diet as an explanatory variable.

The Shannon and Observed species alpha-diversity indices were calculated separately on the data at the ASV level and species level. To bin the ASVs to the species level, *tax_glom()* R function was applied [28]. Comparison of the resulting alpha-diversity figures between the diet groups was done using MWW test. The beta-diversity analysis was performed via principle coordinate analysis (PCoA) on Bray-Curtis dissimilarity matrix, and permutational multivariate analysis of variance (PERMANOVA) test for covariate significance using *adonis()* R function [29], 9999 permutations. The covariates included in the statistical model were the following: diet, sex, pen, and sow.

To screen for bacteria that appeared in higher numbers in one of the feeding groups compared with those of another group, or differentially abundant taxa, the analysis of composition of microbiomes (ANCOM) test was used (false discovery rate (FDR) = 0.05, multiple correction = 2). The test was performed at the phylum, family, and ASV levels [30].

To compare average concentrations of SCFA and to compare colonic CD in the colon between the diet groups, the MWW test was applied.

The loglinear analysis was applied to the histopathology parameter results for comparison between the diet groups. Multiple regression analysis was used to predict the colonic CD. The amplicon sequence variant (ASV) table was transformed to the relative abundances to derive individual bacterial ASV relative abundance figures.

To explore correlations between bacterial group relative abundance and metadata variables, the Pearson’s correlation coefficient (reported as *r*) was applied. To aid the graphical representation of the multiple regression modelling, the numeric variables of the model equation were subjected to principle component analysis (PCA) in R using *prcomp()* function to be further displayed on a biplot. The statistical significance was declared at p-values < 0.05 for all tests.

## Results

### Cultivation results

All the piglets appeared healthy throughout the experiment. There was no mortality, and no difference in feed intake and growth rate between the dietary treatments.

#### Lactobacillus spp.

In the jejunum, LAB were found in higher numbers on average in the yeast group (9.57 logCFU/g) compared with the controls (7.30 logCFU/g) at day 4 PW (*p* < 0.001) (Additional file 1). The same pattern was observed in the ileum at days 4 and 7 PW (yeast = 9.48 vs control = 8.44 logCFU/g, and yeast = 10.0 vs control = 8.61 logCFU/g, respectively) (p < 0.001 for both tests). The variance in the LAB counts in the ileum at days 4, 7 and 14 PW was explained by diet (R^2^_adj_ = 0.45, p < 0.05). Interestingly, when only day 7 was considered, the same linear model could explain 65% of the diet-related variance in the LAB counts. In the cecum and colon, LAB counts were also higher in the yeast group at day 7 PW, and day 14 PW (*p* < 0.05 for both), except in the colon at day 14 PW.

#### Enterococcus spp.

The counts of enterococci (8.98 logCFU/g) were found to be higher in the ileum of the yeast group at day 4 PW compared with those of the controls (8.09 logCFU/g) (p < 0.001). At day 7 PW, enterococci in the colon of the yeast group were higher than those of the control group (9.03 logCFU/g vs 8.20 logCFU/g) (*p* < 0.001).

#### Coliforms

Coliforms were at higher numbers (9.72 logCFU/g) in the cecum of the yeast group at day 7 PW compared with those of the controls (8.47 logCFU/g) (*p* < 0.001).

#### *C. perfringens*

No statistically significant difference was observed between the two feeding groups.

### 16S *rRNA* gene sequencing results

There were on average 449,177 (SD = 57,148) reads per sample after filtering, denoising, and chimeric amplicon removal. There were 2100, 3301, and 3485 ASVs detected in the ileum, cecum, and colon samples, respectively. At day 0 PW, there were 2645 ASVs identified for all sampled gut locations. Similarly, 3378 ASVs and 2994 ASVs were found at day 7 PW and day 14 PW, respectively. The results of sequencing of the positive controls (mock communities) are given in Additional file 2.

### Alpha microbial diversity

**Fig. 2:**
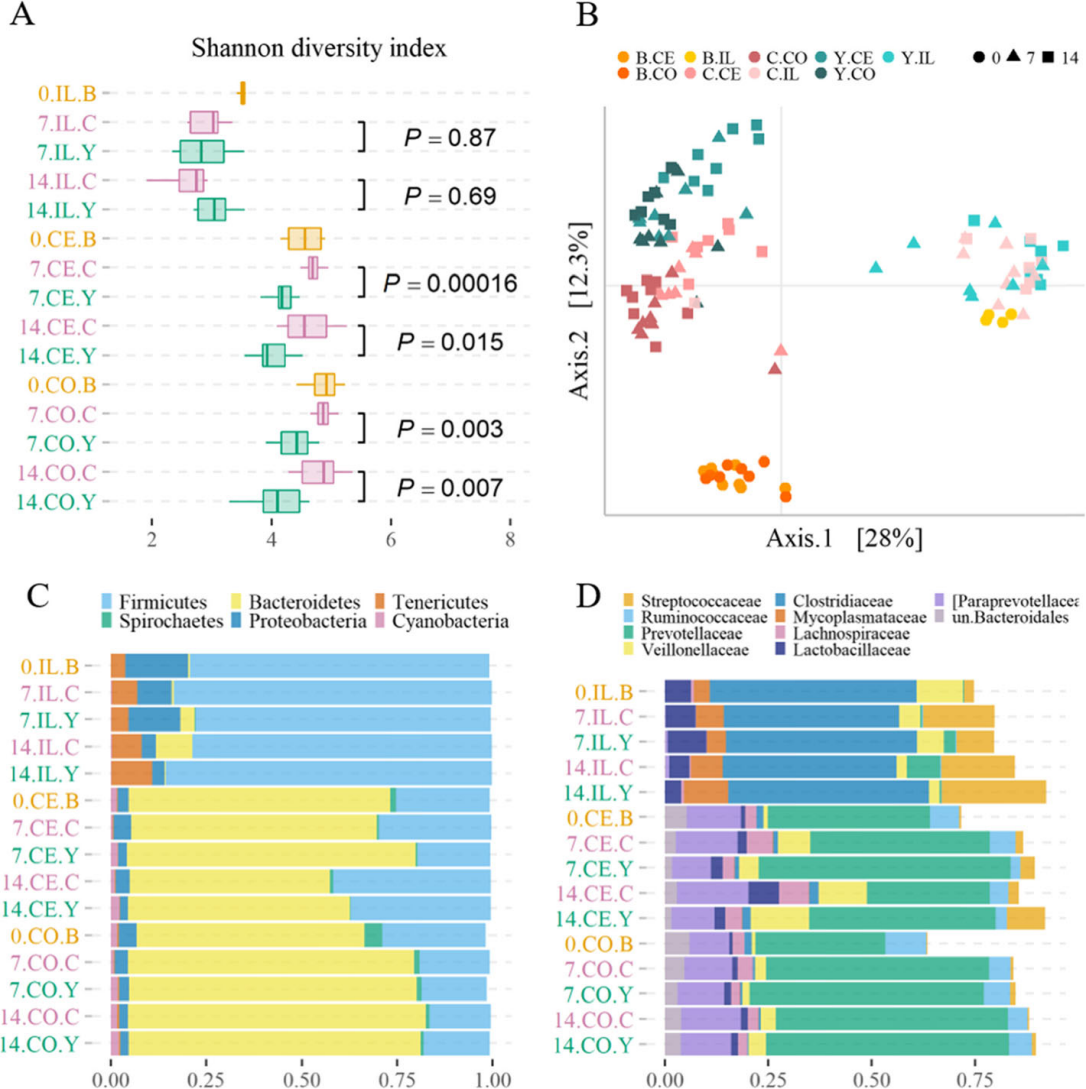
Gut microbiota diversity and composition in postweaning pigs fed with control and yeast diets. Panel **a**: Shannon diversity index in the pig gut microbiota denoted by day, gut site, and diet (**0** day 0 PW, **7** day 7 PW, **14** day 14 PW; **IL** ileum, **CE** caecum, **CO** colon; **B** baseline diet, **C** control diet, **Y** yeast diet) and coloured by diet (baseline *orange*; control *pink*; yeast *dark cyan*). The p-values derive from MWW test comparing the averages of the Shannon diversity index between the diets at each sampling day (n = 8). The box size corresponds to IQRs with the median value represented as the lines inside the box. The whiskers represent upper and lower quartiles of the diversity estimates. Panel **b**: Principal coordinate analysis plot of pig gut microbiota coloured by diet and gut segment (**B.CE** baseline caecum, *orange*; **B.CO** baseline colon, *dark orange*; **B.IL** baseline ileum, *yellow*; **C.CE** controls caecum, *dark pink*; **C.CO** controls colon, *indian red*; **C.IL** controls ileum, *light pink*; **Y.CE** yeast caecum, *dark cyan*; **Y.CO** yeast colon, *teal*; **Y.IL** yeast ileum, *cyan*) and shaped by the day PW (● day 0; ▲ day 7; ■ day 14). Panel **c**: Stacked bar plot showing group average relative abundance of six top abundant bacterial populations at the phylum level in the pig gut denoted by day, gut site, and diet (**0** day 0 PW, **7** day 7 PW, **14** day 14 PW; **IL** ileum, **CE** caecum, **CO** colon; **B** baseline diet, **C** control diet, **Y** yeast diet) and coloured by diet (baseline *orange*; control *pink*; yeast *dark cyan*). The x-axis shows the relative proportions of the bacterial groups coloured with distinct colours. Panel **d**: Stacked bar plot showing group average relative abundance of ten top abundant bacterial populations at the family level in the pig gut denoted as in the panel **c**

The microbial communities in the large intestine of the yeast group were less diverse in comparison to those of the control pigs at day 7 and 14 PW (Fig. 2a, Additional file 3: Table S3) as measured by the Shannon diversity index at the ASV level. There were more distinct ASVs identified in the cecum of the controls than those of the yeast group at day 7 PW and day 14 PW (p < 0.05 for both). There was no difference in alpha microbial diversity when comparing ileum microbiotas between the two diets (Fig. 2a, Additional file 3). Interestingly, when compared at the species level, the Shannon diversity index was higher in cecum microbiota of the controls at day 7 PW only. Otherwise, alpha diversity analysis at the species level showed no difference between the diets (Additional file 3).

### Beta microbial diversity

The PCoA was conducted to compare the gut microbial compositions of individuals by visualizing Bray-Curtis dissimilarity matrices on the plot followed by PERMANOVA statistical test for significance of study covariates. The large intestine microbiotas tended to cluster together according to the diet type (Fig. 2b). At day 7 PW, the diet accounted for 23% of the variance in the large intestine microbial composition (p < 0.001). Even more of the explained variance in microbiota (26%) was attributed to the diet when the large intestine data at day 14 PW were analysed (p < 0.001). Notably, 12% of the variance in the large intestine microbiota was attributed to the sex of the animals at day 7 PW (p < 0.05).

The variance in the ileal microbiota composition was not explained by the dietary treatment, nor due to any other tested covariates (i.e. sex, pen, and sow).

### Distribution of major taxa and differentially abundant taxa

#### Ileum

The ileal microbial consortia primarily consisted of *Firmicutes* (78%), *Proteobacteria* (9%), *Tenericutes* (7%), and *Bacteroidetes* (1%) on average irrespective of the diet (Fig. 2c).

##### Phylum level

There were no differentially abundant phyla identified when comparing the diet groups at any of the sampling days.

##### Family level

The Porphyromonadaceae family was more abundant in the control group compared with that of the yeast group (Additional file 4B).

##### ASV level

Clostridiaceae 02d06 ASV and family *S24* unclassified ASV of Bacteroidales order were found in higher numbers in the control group at day 7 PW compared with those of the yeast group (Additional file 4A). While *Prevotella* ASV9, *Prevotella copri* ASV23 and ASV33, and unclassified ASV of *Lactobacillus* genus were overrepresented in the control ileal microbiota day 14 PW, *Clostridium perfringens* ASV2 and ASV7, and *Lactobacillus salivarius* ASV5 were differentially abundant in the yeast group (Additional file 4B).

#### Caecum

The most abundant phyla in the cecum were the following: *Bacteroidetes* (64%), *Firmicutes* (30%), *Proteobacteria* (3%), and *Cyanobacteria* (1%) (Fig. 2c).

##### Phylum level

**Fig. 3:**
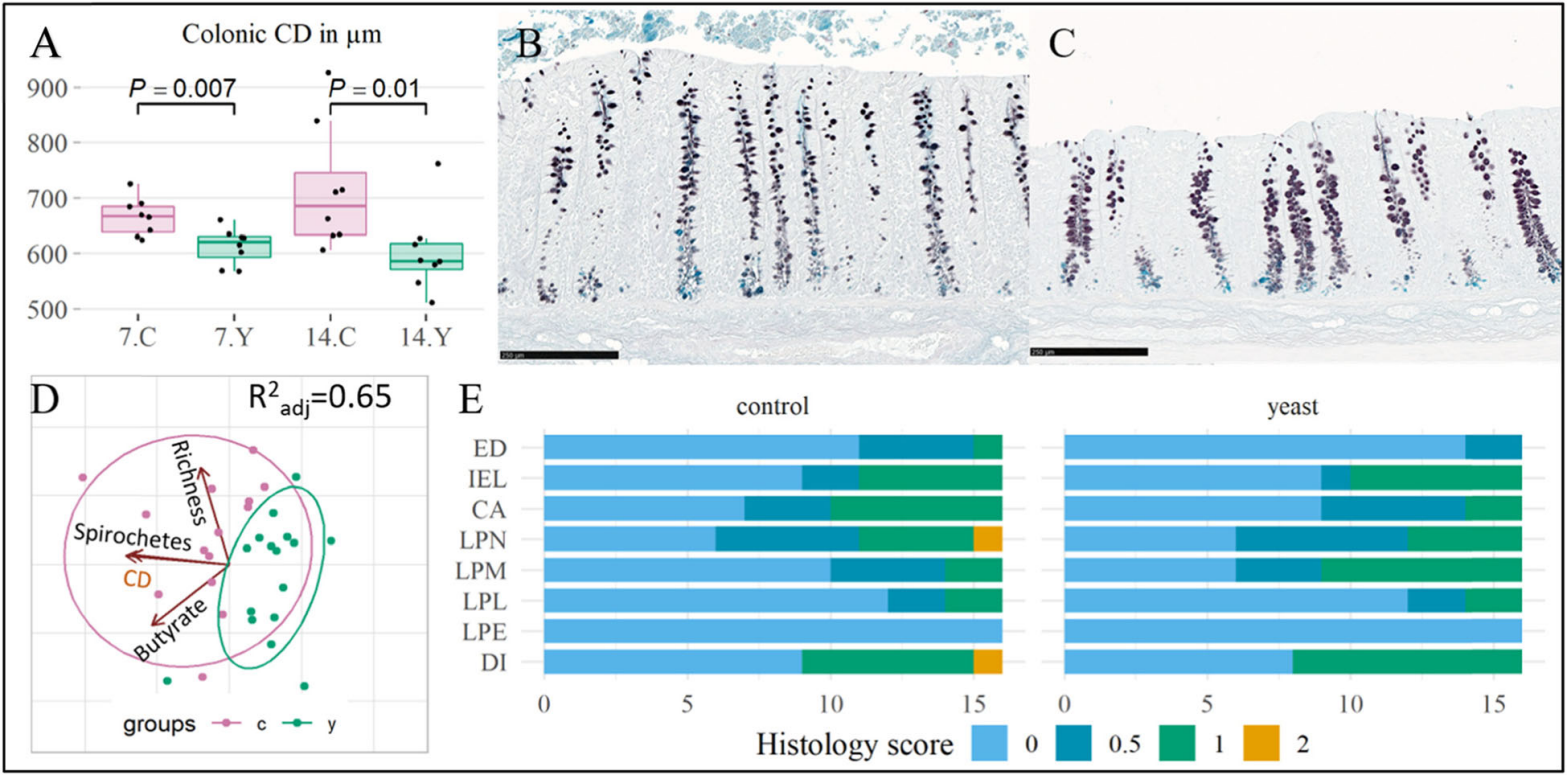
Gut health parameters in postweaning pigs fed with control and yeast diets. Panel **a** Boxplot showing comparison of colonic crypt depth (CD) measurements between the control and yeast group at day 7 PW and day 14 PW coloured and denoted by the diet and day (**7.C** controls day 7 PW *pink*, **7.Y** yeast day 7 PW *dark cyan*, **14.C** controls day 14 PW *pink,***14.Y** yeast day 14 PW *dark cyan*). MWW test p-values are provided above the boxes. Panel **b** Representative section of colon mucosal crypts from the control group at day 14 PW (HID-AB stain, scale bar 250 μm). Panel **c** Representative section of colon mucosal crypts from the yeast group at day 14 PW (HID-AB stain, scale bar 250 μm). Panel **d**: Principal coordinate analysis illustrating contribution of Spirochetes relative abundance, colonic butyrate concentration, and 16S *rRNA* gene richness to the colonic crypt depth. The individual observations and the correlation circles are coloured by the diet (controls *pink*, yeast *dark cyan*). The adjusted R^2^ is given at the right top corner. Panel **e**: Histopathological assessment of the colon mucosa, evaluating epithelial damage (ED), number of intraepithelial lymphocytes (IELs), presence of crypt abscess (CA), and infiltration of leukocytes neutrophils (N), macrophages (M), lymphocytes (L), eosinophils (E), in addition the number of piglets that were diagnosed with a very mild or mild colitis (DI). The horizontal stacked bar plot shows the number of animals with none (0), very mild (1), mild (2), or moderate histopathological changes

There was a higher proportion of *Bacteroidetes* in the yeast group at day 7 PW compared with those of the control group (Fig. 3c, Additional file 4D). Low abundant *Spirochaetes* phylum was overrepresented in the control group at day 14 PW compared with that of the yeast group (Additional file 4E).

##### Family level

An unclassified family of the *Tremblayales* order was found in higher numbers in the control group day 7 when compared with that of the yeast group (Additional file 5C). At day 14 PW, the family s24.7 of *Bacteroides* was more predominant in the control group, whereas an unclassified family of *Alphaproteobacteria* phylum (RF32 order) was overrepresented in the yeast group (Additional file 5D).

##### ASV level

At day 7 PW, three *Prevotella* affiliated amplicons (ASV28, ASV33, ASV50), as well as *Mitsuokella* genus ASV17, were differentially abundant in the yeast group when compared with those of the control group. Conversely, *Faecalibacterium prausnitzii* ASV35 was found to be more abundant in the control group at the same time point (Additional file 5A). Seven different Veillonellaceae family ASVs, including *Selenomonas ruminantium*, *Bulleidia* p.1630.c5, *Parabacteroides*, and four other unclassified taxa were overrepresented in the yeast group at day 14 PW compared with those of the control group. Three variants of unclassified *Prevotella* ASV, *Selenomonas*, *Mitsuokella*, *Mucispirillum schaedleri*, and unclassified ASV of the Porphyromonadaceae family were overrepresented in the yeast group at day 14 PW when compared with those of the control group. An ASV classified as *Selenomonas ruminantium* and *Prevotella, Lactobacillus*, *Campylobacter*, and *Anaerovibrio* ASV33 genera were differentially abundant in the control group at day 14 PW in comparison with those of the yeast group (Additional file 5B).

#### Colon

The colon microbiota structure resembled that of the cecum with *Bacteroidetes* (73%), *Firmicutes* (19%), *Proteobacteria* (3%), *Spirochaetes* (1.8%) and *Cyanobacteria* (1.6%) representing the most dominant phyla (Fig. 2c).

##### Phylum level

There was no statistically significant difference in bacterial phyla abundances between the diet groups at any of the sampling days.

##### Family level

At day 7 PW, more Victivallaceae family ASVs were detected in the control group than in the yeast group (Additional file 6C). At day 14 PW, Succinivibrionaceae and *Bacteroidales* p.2534.18B5 families were found in higher numbers in the control group, whereas Oxalobacteraceae bacterial family was more abundant in the yeast group (Additional file 6D).

##### ASV level

One *Mitsuokella* amplicon variant was more abundant in the yeast group, while an unclassified species of the *Bacteroidales* order (p.2534.18B5 family) was less abundant in the same group at day 7 PW. Two distinct *Prevotella* amplicon variants (ASV2 and ASV50) were differentially abundant in, respectively, control and yeast group. Notably, ASV50, also previously identified in the cecum of the same time point, ranked first on relative abundance, when all samples were considered (Additional file 6A). At day 14 PW, four *Prevotella* ASVs (ASV17, 50, 35, 41), two distinct ASVs classified as *Selenomonas ruminantium* (ASV10, 2), *Mitsuokella* ASV17, *Parabacteroides* ASV22 and ASV23, *Bulleidia* p.1630.c5 and an unclassified ASV of the Veillonellaceae family were found in higher amounts in the yeast group than those in the control group. Four different *Prevotella* amplicons (ASV18, ASV9, 67, 96), *Selenomonas ruminantium* ASV9, *Anaerovibrio* ASV16, and two unclassified ASVs of *Bacteroidales* and YS2 bacterial orders were more abundant in the control group than in the yeast group (Additional file 6B).

### SCFA in the colon

The total colonic SCFA concentration did not differ between the diet groups when measured at day 7 PW (p = 0.32). However, at day 14 PW, the levels of total SCFA tended to be higher in the control group compared with those of the yeast group (p = 0.065). For day 14 PW, butyrate and acetate were found at higher concentration in the control group compared with those of the yeast group (p < 0.05) (Table 2). The concentrations of propionate, valerate, iso-butyrate, and iso-valerate did not differ between the two groups at the statistically significant level.

**Table 2:** Colonic SCFA concentration comparison between the control and the yeast group. The concentration values are reported as μmol/g of colon digesta. Comparison pairs that correspond to p-values less than 0.05 (MWW test) are given in bold

	feed	day 7 PW		day 14 PW	
		mean (SD)	p-value	mean (SD)	*p*-value
**Acetate, μmol/g**	control	57.3 (13.2)	0.156	**60.9 (15)**	0.015
	yeast	46.6 (12.8)		**40.9 (7.07)**	
**Propionate, μmol/g**	control	24.7 (6.9)	0.528	26 (6.31)	0.959
	yeast	25.7 (6.54)		26.3 (9.49)	
**Butyrate, μmol/g**	control	12.08 (4.84)	0.235	**16.5 (4.8)**	0.038
	yeast	9.16 (1.96)		**11.3 (4.52)**	
**Valerate, μmol/g**	control	2.11 (1.0)	0.563	3.12 (1.43)	0.713
	yeast	2.16 (0.6)		3.23 (2.46)	
**Iso-butyrate, μmol/g**	control	0.87 (0.67)	0.558	0.68 (0.24)	0.364
	yeast	0.73 (0.44)		0.81 (0.25)	
**Iso-valerate, μmol/g**	control	0.75 (0.34)	0.242	0.71 (0.31)	0.791
	yeast	0.75 (0.71)		0.83 (0.4)	
**Total SCFA, μmol/g**	control	97.9 (21.8)	0.328	108 (22.3)	0.065
	yeast	85.2 (18.89)		83.5 (20.7)	

### Histology

The colonic CD in the control group was on average deeper than that of the yeast group at both day 7 PW and day 14 PW (p-value = 0.007 and p-value = 0.01, respectively) (Fig. 3a, b, c). The colonic butyrate concentration positively correlated with the crypt depth irrespective of the diet (*r* = 0.55, p-value = 0.001). The prediction model of the colonic CD showed that the depth tended to be deeper with higher numbers of an unclassified Spirochaetaceae ASV (β = 25,500, SE = 5920, t-value = 4.3, p = 0.0002), higher colonic butyrate concentration (β = 6.33, SE = 2.11, t-value = 2.9, p = 0.006), and richer colon microbiota as calculated at ASV level (β = 0.198, SE = 0.094, t-value = 2.1, p = 0.044). Overall the statistical model could be predictive of 65% of variance in colonic CD (Fig. 3d). No statistically significant difference was found in histopathological parameters in the colon comparing the two feeding groups (Fig. 3e).

### Colonic bacteria – colonic butyrate association

The concentration of colonic butyrate positively correlated with the relative abundance of *F. prausnitzii* in the colon (*r* = 0.73, p < 0.0001) (Additional file 7A). The latter accounted for 52% variance in butyrate concentration as estimated by the linear model equation. Inclusion of an unclassified Spirochaetaceae family ASV and *Dialister* genus relative abundance to the existing linear model improved the model with 72% of variance in colonic butyrate production explained Additional file 7B). Notably, the *Oxalobacter* genus, a member of the differentially abundant Oxalobacteriaceae family in the yeast group, negatively correlated with the colon butyrate concentration (*r* = − 0.71, p = 0.002).

### Butyrate – liver – colonic bacteria association

The linear model for predicting the liver index from individual bacterial groups, revealed that *F. prausnitzii* ASV and an unclassified Spirochaetaceae family ASV could explain 47% of variance in the liver index (β = 43.4, SE = 13.3, t-value = 3.2, p = 0.006 and β = 49.7, SE = 22.0, t-value = 2.26, p = 0.04, respectively) (Additional file 8A).

The colon concentration of butyrate positively correlated with the liver index when the two sampling days, day 7 and 14 PW, were considered (*r* = 0.65, p < 0.0001). However, when stratified by diet, the strength of association was different for the control group (*r* = 0.9, p < 0.0001) and the yeast group (*r* = 0.66, p < 0.01) (Additional file 8B, C).

### ADG-butyrate association

At day 7 PW, the relative abundance of the *Proteobacteria* phylum in colon was negatively correlated with ADG (*r* = − 0.65, p = 0.009). The same trend but of lesser magnitude was observed in the ileum and colon samples at both day 7 PW, and day 14 PW (*r* = − 0.37, p = 0.04 and *r* = − 0.41, p = 0.02, respectively). Notably, the relative abundance of the Succinivibrionaceae family was positively correlated with ADG (*r* = 0.47, p = 0.006). A positive correlation was found between the relative abundance of the Prevotellaceae family in the colon of the yeast group and ADG (*r* = 0.53, p = 0.04).

## Discussion

We investigated the effect of a high level *Cyberlindnera jadinii* yeast diet on the gut bacterial compositions in weanling piglets. Protein from *C. jadinii* yeast was used to replace 40% of crude protein in a conventional Norwegian piglet diet. The growth performance and histopathology analysis of the gut tissues indicated that the data obtained was from equally healthy animals. To explore the bacterial composition, 16S *rRNA* gene sequencing and bacterial cultivation were used. The sensitivity of the V1-V3 16S *rRNA* gene amplification assay varied in relation to different bacterial groups. For instance, there was low sensitivity to *Escherichia coli* detection as revealed by the sequencing of a known community standard. With help of cultivation techniques, a fair comparison of coliform numbers between the control and yeast group was obtained. To compare the microbial composition between the diet groups, a phylogeny-agnostic method, permutational multivariate analysis of variance on Bray-Curtis dissimilarity matrix, was used. This decision was based on our observations that the single end sequencing data had provided a limited phylogenetic signal for phylogeny-informed beta diversity method estimates. In contrast, the use of Bray-Curtis dissimilarity matrix was discriminative enough for the comparison of the microbiotas that were presumably similar in their compositions. As expected, the microbiota of the ileum was structurally different from that of the large intestine. Conversely, there was similarity of microbiota compositions between the cecum and colon gut segments. The diets did shape the composition of the large intestine microbiota. The ileum microbiota composition, in contrast, was less affected by diet with only few differences in microbiota at the family and ASV level. This may due to several factors that the host exerts on the bacterial succession in the small intestine. These factors, such as peristalsis, bile acids, pancreatic enzymes, hydrogen ion concentration, and local immunity seem to limit bacterial colonization of the small intestine to those bacterial species recognized by the host immune system [31, 32].

We detected a difference in the alpha bacterial diversity of the large intestine between the yeast and the control groups based on the Shannon index. The control group appeared more diverse at the amplicon level. However, when the bacterial richness and bacterial diversity were examined at the species level, the difference between the diets became less apparent. Only in cecum samples day 7 PW did the diversity figures differ at a statistically significant level. To explain this discrepancy between the analyses done at different resolution levels, namely, ASV and species level, we adopted the following logic; the calculation of bacterial community richness is affected by the number of ASVs inferred from DADA2 pipeline. The more 16S *rRNA* gene amplicon variants detected, the richer the community. On average, the number of the 16S *rRNA* gene copies per genome of the *Firmicutes* (F) tend to be twice as high as that of the *Bacteroidetes* (B) [33]. This estimate prompted us to revisit the difference in F:B ratio at day 7 PW. Indeed, the results of the ANCOM analysis at the phylum level supported that the F:B ratio was higher in the cecum samples of the control group at day 7 PW compared with that of the yeast group. Further, we attempted to verify that it was the intragenomic variability in the number of the 16S *rRNA* gene copies that contributed most to the Shannon diversity calculation. To achieve this, we compared the bacterial richness at the species level between the feeding groups. As expected, there was no difference in species richness between the feeding groups when ASVs were binned at the species level. Therefore, we conclude that the overrepresentation of *Bacteroidetes*-affiliated ASVs in the yeast large intestine resulted in a Shannon diversity that was lower compared with that of the control group. *Firmicutes* may appear in higher numbers in the microbiota of the control pigs because of differences in the diet formulation. In the yeast diet, yeast proteins mostly replaced the conventional protein sources used in the control diet, i.e. soybean meal, potato protein concentrate, fish meal, and rapeseed meal. The presence of various dietary fibres in a diet influences the gut microbiota composition by promoting the growth of *Firmicutes* (reviewed in [34]). It is known that up to 10% of soybean meal, and a considerable portion of rapeseed meal (> 15%) is neutral detergent fibre [7]. Thus, the higher level of soybean- and rapeseed meal in the control diet could account for the high presence of fibre-degrading *Firmicutes* in the large intestine of the control group.

According to our findings, a Prevotellaceae family-related amplicon, ASV50, was predominant in the large intestine of pigs in the yeast group. Even though the analysis of the microbiota composition was confined to sequencing of the 16S *rRNA* bacterial gene only, the overrepresentation of the Prevotellaceae family might be related to the availability of the non-digested parts of the yeast cells in the diet. It is conceivable that the method of yeast processing partially precluded its digestibility in the small intestine thus making the yeast cells available for microbial fermentation in the large intestine. The overgrowth of *Prevotella* in the yeast driven microbiota might also be attributed to the microbial peptidase and proteinase activities of this bacterial groups [35]. Previous studies by Mach et al. [36] and by Ramayo-Caldas et al. [37], showed that the enterotype dominated by *Prevotella* and *Mitsuokella* species is associated with lowered alpha diversity and improved growth performance. These findings are in line with our results on alpha bacterial diversity and ADG in the yeast group. The lower levels of butyrate and acetate in the colon of the yeast fed piglets may be due to the predominance of the *Prevotella-Mitsuokella*-affiliated groups and hence a suppression of certain SCFA-producers [37]. Higher abundance of *Mitsuokella* in the large intestine of yeast fed animals is consistent with the studies where yeast was supplemented [8]. Furthermore, in a study using similar dietary formulations as the present study, Cruz and co-workers [7] showed that the total tract digestibility of phosphorous was higher in the yeast group than in the control group. As *Mitsuokella* and *Selenomonas* genera are reported to release phosphorous from phytate [38, 39], it is tempting to ascribe this metabolic activity to these bacteria. The *Mitsuokella* and *Selenomonas* genera were found in higher numbers in the yeast group compared with the numbers in the control group. However, the resolution of the 16S *rRNA* gene method does not always provide enough confidence in assigning the PCR amplicons to the species level. To learn about the functional potential and contribution to host metabolism of the mentioned Selenomonadaceae and Prevotellaceae, use of anaerobic cultivation techniques may be necessary. It has previously been reported that in gut eco-systems supplied with low levels of yeast-derived components, the outgrowth of *Prevotella*, *Selenomonas*, and *Mitsuokella* commonly co-occurs with the reduction in SCFA producing bacteria [9–11]. The interpretation of the ANCOM analysis revealed more *F. prausnitzii* (97.5% identity to *F. prausnitzii* strain ATCC 27768, GenBank accession: NR_028961) in the caecum of the control group than that of the yeast group. To our knowledge, the ANCOM test we used for identification of differentially abundant taxa between the two dietary groups performs better than other tests with respect to false discovery rate control. However, when applied to groups with less than twenty samples per group, the sensitivity of ANCOM decreases [40]. In our 16S *rRNA* gene sequencing setup, *F. prausnitzii* on average represented 0.9% of the caecum microbiota population and 0.3% of the colon microbiota population. It is possible that eight samples per group were not enough for ANCOM to detect differences in rare colonic *F. prausnitzii* between the two groups. However, irrespective of the dietary interventions, we found that *F. prausnitzii* was positively correlated with the colonic butyrate concentration. The opposite relationship was found for the *Oxalobacter* genus. *Oxalobacter* strain OxB, an oxalate degrader, was studied by Allison et al. [41]. Allison and co-workers showed that acetate is an essential nutrient for growth of the bacterium. Their findings suggest that a competition for the nutrient between *F. prausnitzii* and *Oxalobacter* is conceivable [41, 42]. There was a mutual exclusion relationship between the two bacteria at a statistically significant level when examined with the CoNet co-occurrence network analysis [43] (data not shown). In addition to the role of *F. prausnitzii* in butyrate production, our results suggest that *Dialister* and an unclassified member of the Spirochaetaceae family may contribute to the colonic butyrate pool.

Next, we have found an association between the concentration of colonic butyrate and the liver index. Similarly, the number of *F. prausnitzii* in the colon correlated with the liver index. The portal vein concentration of butyrate is known to reflect the production levels of butyrate in the colon [44]. Thus, it is likely that the liver index was related to the uptake and metabolism of butyrate in the liver in our study. It is intriguing to think that gut microbiota members may be involved in the butyrate metabolism to the extent where the size of the liver is affected. Reduced level of butyric acid has been shown to be associated with pathologic conditions in man [45]. Butyrate has been implicated to play a role in the integrity of the intestinal wall, serving as an energy source for colonocytes and as a regulatory molecule [46, 47]. However, it is unclear what concentration of butyrate is optimal to maintain gut integrity and homeostasis in weanling piglets. Furthermore, butyrate has been shown to impact actively on the colonic crypt stem cells [18, 19]. Wang and co-workers demonstrated that butyrate diminished the crypt cell proliferation in a dose-response manner in an in vitro human colon crypt array [17]. In the present study, we observed a difference in the colonic CD between the two diet groups that possibly could be attributed to the altered abundance of butyrate-producing bacteria. Similar findings but of a lesser magnitude were reported by Mentschel and Claus in a study where piglets were fed with resistant potato starch [18]. In the light of our findings and previous publications [17–19], there is a good reason to believe that crypt elongation is a compensatory change to protect the crypt stem cell compartment from butyrate toxicity. The correlation between the colonic butyrate and the liver size suggests that the colonocytes received butyrate levels exceeding their metabolic capacity, with the butyrate surplus being transported to the liver. Histopathological examination of intestinal tissues did not reveal any difference in gut health parameters between the two feeding groups. Thus, it is tempting to speculate that there is a saturation point in the butyrate microbial production beyond which butyrate is not required as a fuel for colonocytes.

The cultivation results demonstrated that overall the differences between the feeding groups in the counts of LAB, enterococci, and coliforms were detected from day 7 PW. Much of the inter-individual variation before day 7 PW may be attributed to the weaning event. The bacterial succession of the gut is governed by, but not limited to, the substrate availability, gut physiology, and immune status. Feed intake during the time of weaning, when the piglets shift from milk to solid feed, is a key factor for immune system maturation [22], and luminal wall development [21]. The weaning event entails an irregular and variable timing in the acceptance of the new type of diet. This, consequently, leads to a transient starvation in some animals. According to our observations, albeit non-systematic, this was the case in our experiment. To this end, it is to be expected that the major variability in bacterial succession occurs during the first two weeks PW. LAB were consistently found in higher numbers in the ileum and large intestine of the yeast group compared with intestines of the control group. This bacterial group has a range of bioactive properties known to benefit mammals (reviewed in [48]). Attempts have been made to graft LAB into GI tract to improve health or ameliorate disease [49]. The ileum has a very dynamic gut environment, where bacteria must overcome multiple factors (e.g. digesta flow, peristalsis, microbe-host interaction, and microbe-microbe interaction) if they are to colonize and persist in the intestinal segment [32]. It has been reported that LAB are capable of adhesion to the intestinal cell wall [48]. Russo et al. showed that adhesion to human enterocytes of some LAB strains in vitro was inducible by β-D-glucan extracted from *Pediococcus parvulus* [50]. Therefore, the higher abundance of intestinal LAB in the yeast group in the present study may be attributable to the presence of a β-glucan fraction from the yeast cell wall in the feed. It is also tempting to speculate that the presence of yeast cell wall glucans in the feed affects digesta viscosity in the lumen, which is a factor that would favour LAB colonization. Snart and colleagues demonstrated that high-viscosity dietary fibre β-glucans supplementation was positively associated with higher numbers of lactobacilli in the caecum of rats [51]. Supplementation with the yeast cell wall was implicated in an increase in lactobacilli numbers in the ileal digesta of broilers in the studies by Liy et al. and Ghosh et al. [52, 53]. Their findings suggest that the yeast cell wall, or its components, may have selective properties towards LAB in a range of hosts. It is notable that *L. salivarius* 16S *rRNA* gene amplicon relative abundance was found in higher numbers in the ileum of the pigs fed yeast at day 14 PW compared with that of the control pigs. The concordance between the results obtained from culture-dependent and -independent methods strengthens the validity of our findings. The augmentation of intestinal LAB is a promising aspect of the yeast-derived diet in GI tract of pigs. However, further research is needed to elucidate whether it is the yeast wall β-glucans or other ingredients of the diet that favour the LAB increase.

## Conclusions

The replacement of 40% of the crude protein from the main protein sources traditionally used in Norway with proteins from *Cyberlindnera jadinii* in a weanling piglet diet reshaped the large intestine microbiota structure. The microbiota of yeast fed piglets showed a dominance of *Prevotella*-, *Mitsuokella*- and *Selenomonas*-related taxa along with the decreased alpha-diversity. Larger numbers of viable LAB cells were recovered from both small and large intestines of the yeast fed piglets compared with the control piglets. Owing to the functional capacity of the above bacterial groups, we believe that *Cyberlindnera jadinii* yeast, in addition of being a high-quality protein source, promote growth of beneficial gut microbes.

## Supplementary information

**Additional file 1.** Comparison of bacterial CFUs on the selective agar plates. The values are the group medians (IQR) of logCFUs per gram of lumen contents. The bold font indicates statistically significant level (p < 0.05) of MWW test.

**Additional file 2.** Relative abundance of bacterial genera in the sequenced mock community standards along with their expected abundance.

**Additional file 3.** Alpha bacterial diversity as measured by a) Observed species and b) Shannon diversity index. The average values between the control and yeast group are represented as mean values with standard deviation (SD) along with medians with inter-quartile ranges (IQR). Comparison pairs that correspond to p-values less than 0.05 (MWW test) are given in bold.

**Additional file 4.** Differentially abundant ASVs between the yeast and control diets (ileum, caecum). A ileum, d 7 PW, B ileum, d 14 PW, C ileum, d 14 PW (family level), D caecum, d 7 PW (phylum level), E caecum, d 14 PW (phylum level). All taxonomic entities appeared as differentially abundant at FDR = 0.05.

**Additional file 5.** Differentially abundant ASVs between the yeast and control diets (caecum). A, caecum, d 7 PW, B caecum, d 14 PW, C caecum, d 7 PW (family level), D caecum, d 14 PW (family level). All taxonomic entities appeared as differentially abundant at FDR = 0.05.

**Additional file 6.** Differentially abundant ASVs between the yeast and control diets (colon). A colon, d 7 PW, B colon, d 14 PW, C colon, d 7 PW (family level), D colon, d 14 PW (family level). All taxonomic entities appeared as differentially abundant at FDR = 0.05.

**Additional file 7. **Association of colonic butyrate concentration with individual bacterial groups. Panel A: Correlation plot of colonic butyrate concentration (measured in μM per gram of intestinal contents) against *F. prausnitzii* relative abundance measured at days 7 and 14 PW (n = 32). The dots are coloured by the diet (control *pink*; yeast *dark cyan*). Pearson’s *rho* is reported above the regression line. Panel B: Principal component analysis performed on the relative abundance of Spirochaetaceae, Faecalibacterium, Dialister and molarities of butyrate in the colon of pigs measured at days 7 and 14 PW (n = 32 but 3 dots are not shown). The dots are coloured by the diet (control *pink*; yeast *dark cyan*). The vectors represent the degree of correlation between the bacterial groups data and the butyrate concentration data.

**Additional file 8. **Association of liver index with individual bacterial groups, and colonic butyrate concentration. Panel A: Principal component analysis performed on the relative abundance of Spirochaetaceae, Faecalibacterium and the liver index of pigs measured at days 7 and 14 PW (n = 32 but 2 dots are not shown). The dots are coloured by the diet (control *pink*; yeast *dark cyan*). The vectors represent the degree of correlation between the bacterial groups data and the liver index data. Panel B: Correlation plot of colonic butyrate concentration (measured in μM per gram of intestinal contents) against liver index measured in the control group pigs at days 7 and 14 PW (n = 16). The dots are coloured by the diet (control *pink*). Pearson’s *rho* is reported above the regression line. Panel C: Correlation plot of colonic butyrate concentration (μM/gram of intestinal contents) against liver index measured in the yeast group pigs at days 7 and 14 PW (n = 16). The dots are coloured by the diet (yeast *dark cyan*). Pearson’s *rho* is reported above the regression line.

## Data Availability

The raw sequencing reads are deposited in the SRA archive: PRJNA580284.

## Abbreviations

16S *rRNA*: 16S ribosomal ribonucleic acid
ADG: Average daily gain
ANCOM: Analysis of compositions of microbiomes
ASV: Amplicon sequence variant
CD: Crypt depth
CFU: Colony forming unit
DNA: Deoxyribonucleic acid
HID-AB: High iron diamine-alcian blue
LAB: Lactic acid-producing bacteria
MWW: Mann-Whitney-Wilcoxon
PERMANOVA: Permutational multivariate analysis of variance
PW: Post-weaning
PCoA: Principle coordinate analysis
PCA: Principle component analysis
SCFA: Short-chain fatty acid
SE: Standard error
SRA: Sequence Read Archive
