# Supplementary material for: Effect of Cyberlindnera jadinii yeast as a protein source on intestinal microbiota and butyrate levels in post-weaning piglets
Source: Anim Microbiome. 2020 May 5;2:13. doi: 10.1186/s42523-020-00031-x (PMC7807459; doi:10.1186/s42523-020-00031-x)
Supplement: Supplementary file 1 — Additional file 1. Comparison of bacterial CFUs on the selective agar plates. The values are the group medians (IQR) of logCFUs per gram of lumen contents. The bold font indicates statistically significant level (p < 0.05) of MWW test. [file 42523_2020_31_MOESM1_ESM.docx]

|  | D0 | D2 | | D4 | | D7 | | D14 | |
| --- | --- | --- | --- | --- | --- | --- | --- | --- | --- |
|  | baseline | control | yeast | control | yeast | control | yeast | control | yeast |
|  | ***C. perfringens*** | | | | | | | | |
| jejunum | 7.36(1.1) | 6.90(1.1) | 6.62(0.3) | 5.89(0.2) | 7.30(0.5) | 6.14(0.2) | 6.07(0.8) | 6.88(0.7) | 6(0.5) |
| ileum | 8.46(1.8) | 7.30(0.2) | 7.01(0.9) | 6.04(0.4) | 7.64(1.8) | 6.63(0.3) | 6.66(0.2) | 6.30(1.4) | 6.70(0.9) |
| cecum | 8.27(0.7) | 8.64(1.3) | 8.78(1.2) | 8.07(1.4) | 8.66(0.6) | 8.89(0.9) | 9.10(0.9) | 8.73(0.8) | 9.38(0.8) |
| colon | 8.68(0.8) | 8.51(0.6) | 9.44(0) | 8.88(0.4) | 8.98(0.6) | 9.55(0.7) | 9.40(0.6) | 9.02(1) | 9.38(0.3) |
|  | **Coliforms** | | | | | | | | |
| jejunum | 7.32(0.3) | 7.46(0.8) | 7.75(1.5) | 8.01(1.3) | 8.48(0.6) | 7.77(1.1) | 7.23(1.1) | 6.15(0.6) | 6.47(0.2) |
| ileum | 9.82(0.8) | 9.23(1.3) | 9.36(1.2) | 9.53(0.7) | 9.83(0.5) | 9.17(1.5) | 10.2(1.4) | 7.32(0.8) | 7.57(0.8) |
| cecum | 10.0(1.3) | 9.16(1) | 9.45(1.1) | 9.93(0.8) | 9.51(0.9) | **8.47(0.6)** | **9.72(1.3)** | 7.91(0.9) | 8.13(0.5) |
| colon | 9.90(0.9) | 9.63(0.5) | 9.62(0.6) | 9.87(1.3) | 9.34(0.8) | 8.85(1.2) | 9.48(0.5) | 7.77(1) | 7.94(0.4) |
|  | **Enterococci** | | | | | | | | |
| jejunum | 9.16(0.4) | 8.28(1.3) | 8.75(1) | 7.23(0.2) | 8.17(3) | 7.38(1.4) | 7.54(0.5) | 8.25(1.3) | 7.14(3) |
| ileum | 10.0(0.1) | 9.39(1.5) | 10.0(0.6) | **8.09(1.4)** | **8.98(1.5)** | 7.88(0.6) | 9.10(0.7) | 7.60(1.5) | 6.46(0.3) |
| cecum | 9.88(0.8) | 9.80(0.7) | 9.80(0.6) | 9.34(0.2) | 9.66(1.1) | 8.35(0.9) | 9.10(0.5) | 6.43(1.3) | 8.03(1.7) |
| colon | 9.73(0.9) | 9.99(1) | 9.85(0.8) | 9.53(0.5) | 9.29(0.3) | **8.20(0.8)** | **9.03(0.5)** | 9.07(2) | 7.25(1.4) |
|  | **LAB** | | | | | | | | |
| jejunum | 9.07(0.3) | 8.81(2.6) | 9.15(0.7) | **7.30(0.3)** | **9.57(0.6)** | 8.43(0.2) | 8.38(0.6) | NA | 7.73(0.6) |
| ileum | 10.1(0.4) | 9.60(0.8) | 9.94(0.4) | **8.44(0.5)** | **9.48(1.4)** | **8.61(0.3)** | **10.0(0.5)** | 8(0.2) | 8.78(0.2) |
| cecum | 10.0(0.8) | 9.99(0.9) | 9.52(0.6) | 9.41(0.2) | 9.99(1.3) | **8.57(0.4)** | **9.49(0.3)** | **8.38(0.8)** | **9.27(0.3)** |
| colon | 10.0(1.3) | 9.37(0.6) | 9.68(1.2) | 9.36(0.9) | 9.65(0.8) | **8.86(0.6)** | **9.81(0.6)** | 8.79(0.7) | 8.81(1.6) |
