# Supplementary material for: Effect of Cyberlindnera jadinii yeast as a protein source on intestinal microbiota and butyrate levels in post-weaning piglets
Source: Anim Microbiome. 2020 May 5;2:13. doi: 10.1186/s42523-020-00031-x (PMC7807459; doi:10.1186/s42523-020-00031-x)
Supplement: Supplementary file 2 — Additional file 2. Relative abundance of bacterial genera in the sequenced mock community standards along with their expected abundance. [file 42523_2020_31_MOESM2_ESM.docx]

| Genera | run #1 relative abundance | run #2 relative abundance | expected relative abundances |
| --- | --- | --- | --- |
| *Staphylococcus* | 30.234% | 31.691% | 19.8% |
| *Streptococcus* | 28.531% | 29.124% | 19.8% |
| *Porphyromonas* | 28.353% | 26.880% | 18.0% |
| *Rhodobacter* | 1.598% | 1.818% | 18.0% |
| *Escherichia/Shigella* | 0.946% | 0.649% | 18.0% |
| *Clostridium*_sensu_stricto_1 | 6.756% | 6.923% | 1.8% |
| *Bacillus* | 2.491% | 1.903% | 1.8% |
| *Pseudomonas* | 0.053% | 0.036% | 1.8% |
| *Lactobacillus* | 0.329% | 0.334% | 0.18% |
| *Neisseria* | 0.263% | 0.246% | 0.18% |
| *Helicobacter* | 0.128% | 0.149% | 0.18% |
| *Cutibacterium* | 0.123% | 0.131% | 0.18% |
| *Acinetobacter* | 0.012% | 0.009% | 0.18% |
| *Bacteroides* | 0.051% | 0.048% | 0.02% |
| *Enterococcus* | 0.019% | 0.015% | 0.02% |
| *Deinococcus* | 0.014% | 0.016% | 0.02% |
| *Actinomyces* | 0.011% | 0.013% | 0.02% |
| *Bifidobacterium adolescentis* | 0.000% | 0.000% | 0.02% |
