# Supplementary material for: Effect of Cyberlindnera jadinii yeast as a protein source on intestinal microbiota and butyrate levels in post-weaning piglets
Source: Anim Microbiome. 2020 May 5;2:13. doi: 10.1186/s42523-020-00031-x (PMC7807459; doi:10.1186/s42523-020-00031-x)
Supplement: Supplementary file 3 — Additional file 3. Alpha bacterial diversity as measured by a) Observed species and b) Shannon diversity index. The average values between the control and yeast group are represented as mean values with standard deviation (SD) along with medians with inter-quartile ranges (IQR). Comparison pairs that correspond to p-values less than 0.05 (MWW test) are given in bold. [file 42523_2020_31_MOESM3_ESM.docx]

|  | Observed | | | | | | | | | | |
| --- | --- | --- | --- | --- | --- | --- | --- | --- | --- | --- | --- |
|  |  | Amplicon level | | | | | Species level | | | | |
|  | day PW | 0 | 7 | | 14 | | 0 | 7 | | 14 | |
|  | diet | baseline | control | yeast | control | yeast | baseline | control | yeast | control | yeast |
| Ileum | mean (SD) | 215.5 (34.92) | 219.5 (78.35) | 260.4 (198.7) | 296.2 (96.89) | 212.4 (76.2) | 71.25 (11.73) | 85.88 (21.81) | 83.29 (40.32) | 99.5 (18.07) | 90.57 (20.99) |
|  | median (IQR) | 225 (30.5) | 225 (108) | 219 (261) | 299.5 (129.25) | 175 (57.5) | 69.5 (17.25) | 92 (33) | 93 (54) | 103 (22.75) | 83 (19.5) |
|  | p-value |  | 0.86 | | 0.072 | |  | 0.86 | | 0.41 | |
| Cecum | mean (SD) | 700.6 (70.92) | **658.5 (69.82)** | **572.1 (61.83)** | **672.4 (120.1)** | **525.8 (87.88)** | 111.5 (5.71) | 106.75 (10.78) | 105.5 (10.46) | 109.25 (8.08) | 106.12 (7.99) |
|  | median (IQR) | 686 (69.25) | **677.5 (51.75)** | **580 (56)** | **659.5 (150.25)** | **544.5 (143)** | 112.5 (4.25) | 105 (16.5) | 106 (17) | 111.5 (5.75) | 108 (9.75) |
|  | p-value |  | **0.015** | | **0.027** | |  | 0.792 | | 0.34 | |
| Colon | mean (SD) | 855.2 (111.74) | 741 (86.61) | 657 (77.97) | 684.1 (131.97) | 563.1 (99.83) | 119.88 (6.88) | 112.25 (7.96) | 108.62 (7.48) | 105.5 (8.32) | 103.5 (6.91) |
|  | median (IQR) | 829.5 (193) | 750.5 (95.5) | 663 (85.25) | 725.5 (117.75) | 555.5 (135.75) | 117 (10.25) | 109.5 (8.25) | 109.5 (7) | 106 (10.25) | 103.5 (8) |
|  | p-value |  | 0.06 | | 0.06 | |  | 0.63 | | 0.83 | |
|  | Shannon diversity | | | | | | | | | | |
|  |  |  |  |  |  |  |  |  |  |  |  |
|  |  | Amplicon level | | | | | Species level | | | | |
|  | day PW | 0 | 7 | | 14 | | 0 | 7 | | 14 | |
| Ileum | diet | baseline | control | yeast | control | yeast | baseline | control | yeast | control | yeast |
|  | mean (SD) | 3.59 (0.07) | 2.59 (0.87) | 2.92 (0.49) | 2.86 (0.78) | 2.7 (0.85) | 2.25 (0.12) | 1.9 (0.57) | 1.97 (0.67) | 1.97 (0.3) | 1.91 (0.49) |
|  | median (IQR) | 3.59 (0.06) | 2.9 (0.77) | 2.84 (0.75) | 2.77 (0.38) | 3.04 (0.98) | 2.24 (0.17) | 2.07 (0.5) | 2.07 (0.89) | 1.99 (0.33) | 2.18 (0.78) |
|  | p-value |  | 0.86 | | 0.69 | |  | 0.86 | | 0.95 | |
| Cecum | mean (SD) | 4.65 (0.3) | **4.82 (0.17)** | **4.29 (0.22)** | **4.72 (0.44)** | **4.1 (0.32)** | 2.79 (0.25) | **2.73 (0.2)** | **2.34 (0.19)** | 2.92 (0.21) | 2.65 (0.2) |
|  | median (IQR) | 4.64 (0.49) | **4.79 (0.18)** | **4.27 (0.21)** | **4.65 (0.6)** | **4.02 (0.34)** | 2.79 (0.4) | **2.75 (0.26)** | **2.34 (0.33)** | 2.85 (0.2) | 2.57 (0.33) |
|  | p-value |  | **0.0001** | | **0.007** | |  | **0.001** | | 0.065 | |
| Colon | mean (SD) | 4.93 (0.27) | **4.94 (0.21)** | **4.48 (0.33)** | **4.89 (0.38)** | **4.17 (0.46)** | 3.07 (0.22) | 2.67 (0.19) | 2.48 (0.31) | 2.55 (0.3) | 2.41 (0.34) |
|  | median (IQR) | 4.94 (0.35) | **4.96 (0.19)** | **4.52 (0.45)** | **4.94 (0.51)** | **4.16 (0.62)** | 3.14 (0.18) | 2.69 (0.16) | 2.41 (0.44) | 2.5 (0.35) | 2.35 (0.36) |
|  | p-value |  | **0.004** | | **0.01** | |  | 0.19 | | 0.44 | |
