# Supplementary figures and images for: Effect of Cyberlindnera jadinii yeast as a protein source on intestinal microbiota and butyrate levels in post-weaning piglets
Source: Anim Microbiome. 2020 May 5;2:13. doi: 10.1186/s42523-020-00031-x (PMC7807459; doi:10.1186/s42523-020-00031-x)

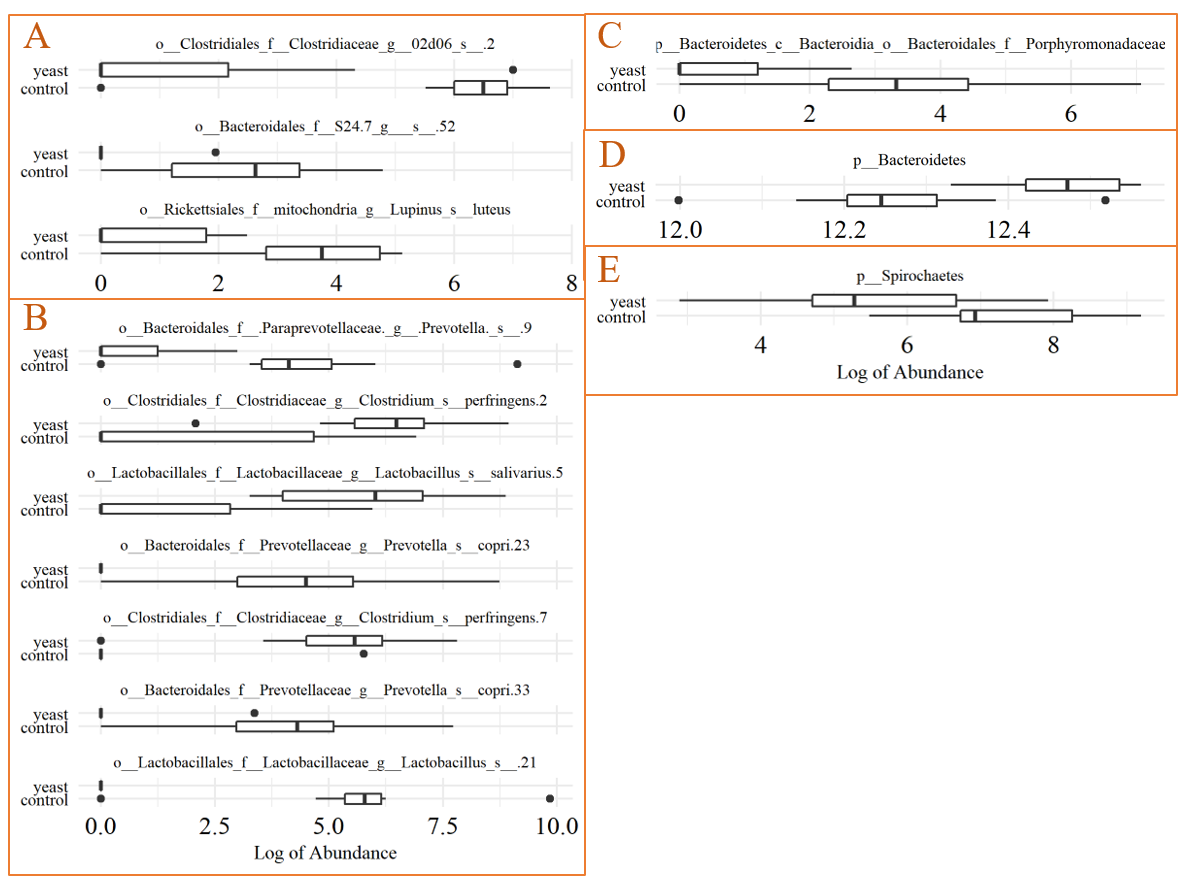

Supplement: Supplementary file 4 — Additional file 4. Differentially abundant ASVs between the yeast and control diets (ileum, caecum). A ileum, d 7 PW, B ileum, d 14 PW, C ileum, d 14 PW (family level), D caecum, d 7 PW (phylum level), E caecum, d 14 PW (phylum level). All taxonomic entities appeared as differentially abundant at FDR = 0.05. [file 42523_2020_31_MOESM4_ESM.docx]

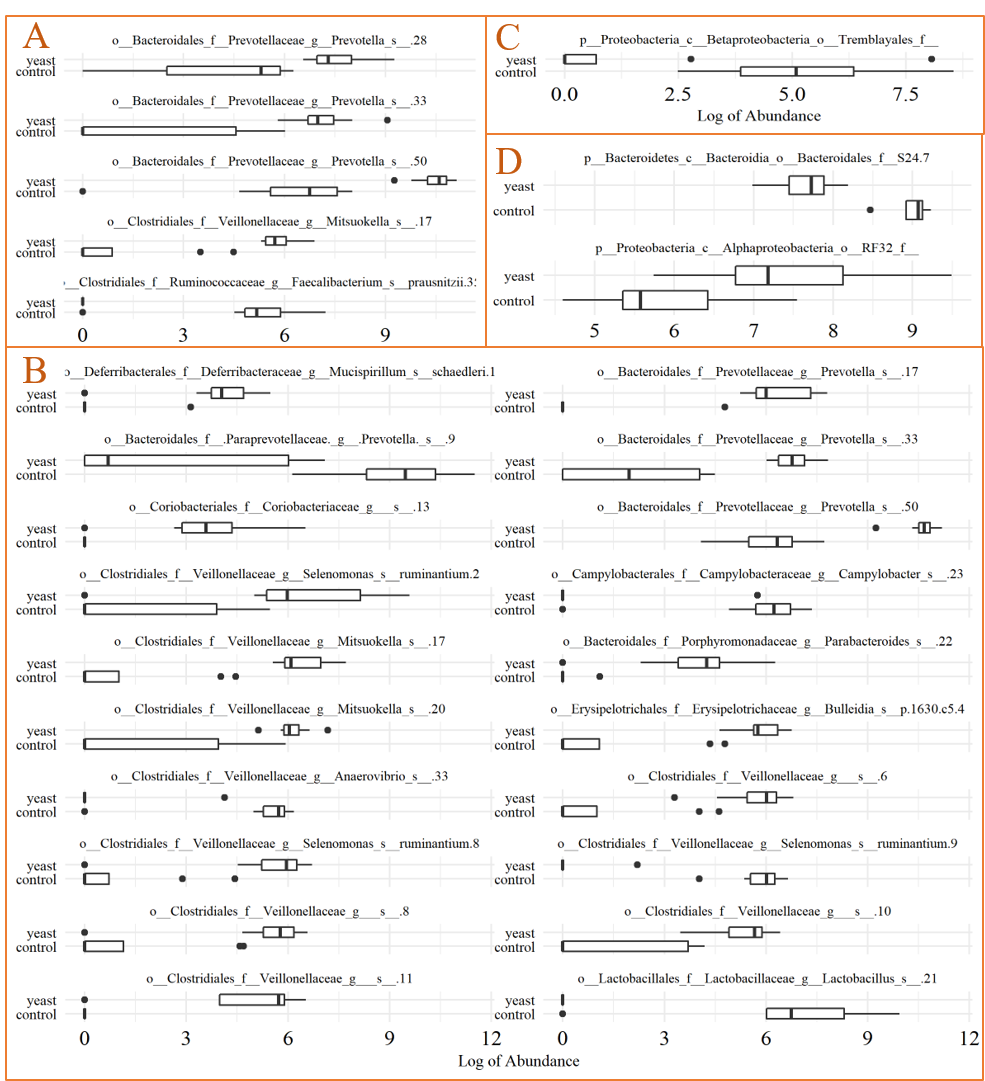

Supplement: Supplementary file 5 — Additional file 5. Differentially abundant ASVs between the yeast and control diets (caecum). A, caecum, d 7 PW, B caecum, d 14 PW, C caecum, d 7 PW (family level), D caecum, d 14 PW (family level). All taxonomic entities appeared as differentially abundant at FDR = 0.05. [file 42523_2020_31_MOESM5_ESM.docx]

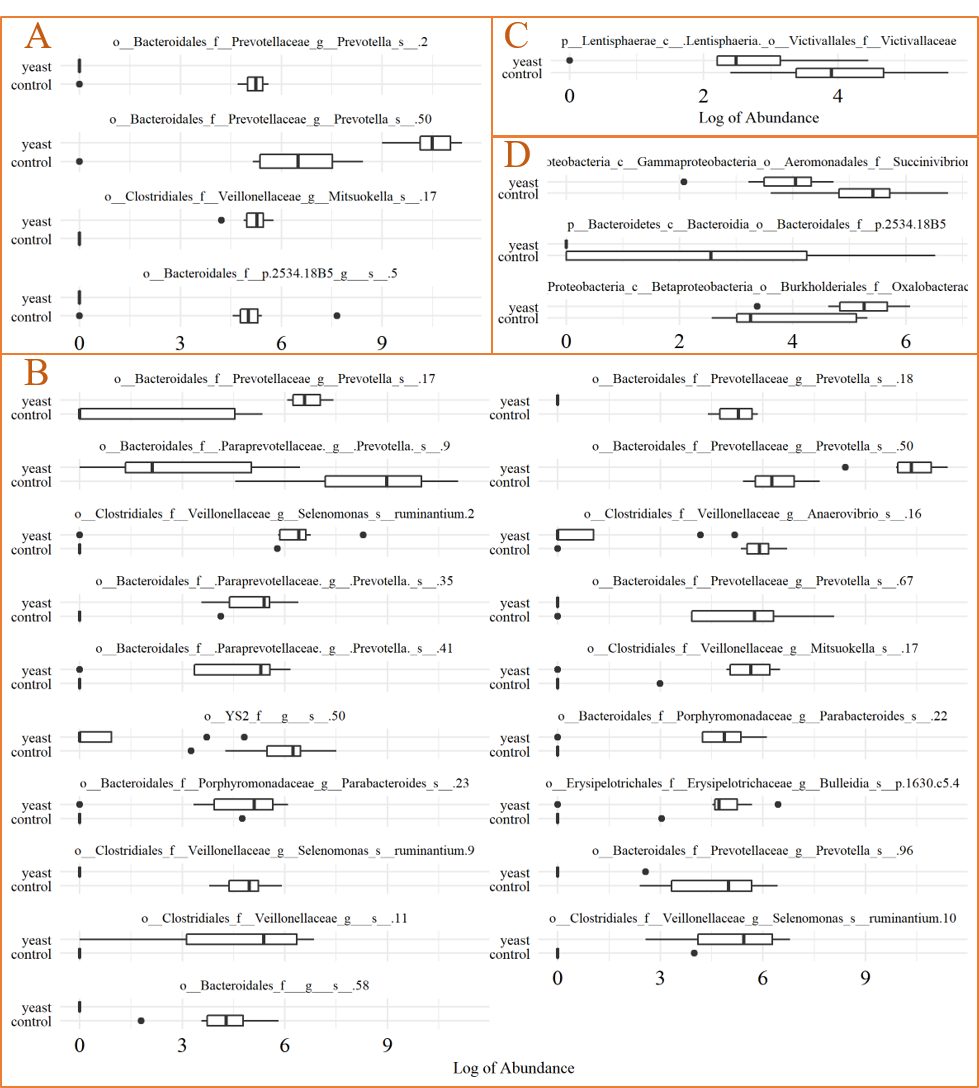

Supplement: Supplementary file 6 — Additional file 6. Differentially abundant ASVs between the yeast and control diets (colon). A colon, d 7 PW, B colon, d 14 PW, C colon, d 7 PW (family level), D colon, d 14 PW (family level). All taxonomic entities appeared as differentially abundant at FDR = 0.05. [file 42523_2020_31_MOESM6_ESM.docx]

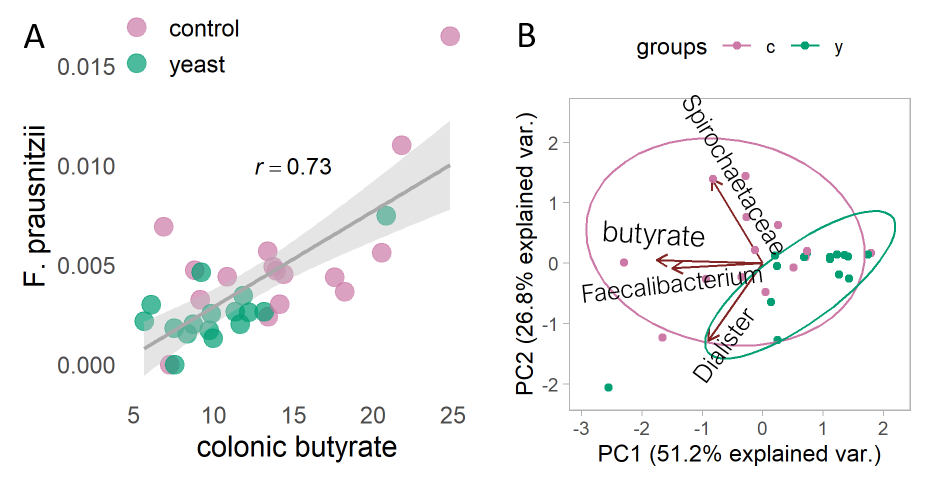

Supplement: Supplementary file 7 — Additional file 7. Association of colonic butyrate concentration with individual bacterial groups. Panel A: Correlation plot of colonic butyrate concentration (measured in μM per gram of intestinal contents) against F. prausnitzii relative abundance measured at days 7 and 14 PW (n = 32). The dots are coloured by the diet (control pink; yeast dark cyan). Pearson’s rho is reported above the regression line. Panel B: Principal component analysis performed on the relative abundance of Spirochaetaceae, Faecalibacterium, Dialister and molarities of butyrate in the colon of pigs measured at days 7 and 14 PW (n = 32 but 3 dots are not shown). The dots are coloured by the diet (control pink; yeast dark cyan). The vectors represent the degree of correlation between the bacterial groups data and the butyrate concentration data. [file 42523_2020_31_MOESM7_ESM.docx]

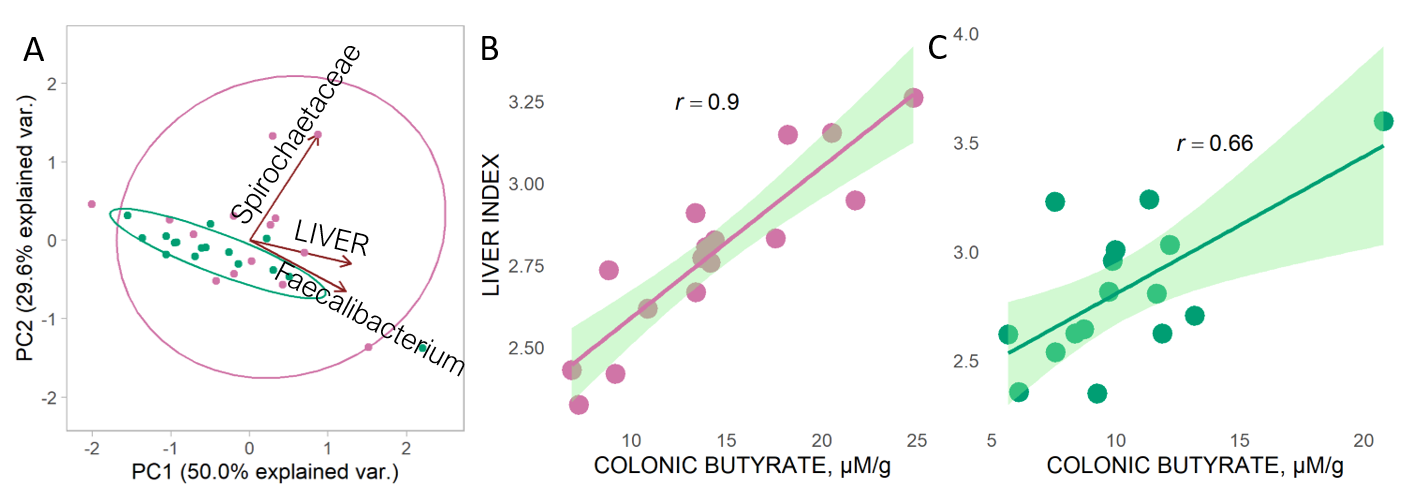

Supplement: Supplementary file 8 — Additional file 8. Association of liver index with individual bacterial groups, and colonic butyrate concentration. Panel A: Principal component analysis performed on the relative abundance of Spirochaetaceae, Faecalibacterium and the liver index of pigs measured at days 7 and 14 PW (n = 32 but 2 dots are not shown). The dots are coloured by the diet (control pink; yeast dark cyan). The vectors represent the degree of correlation between the bacterial groups data and the liver index data. Panel B: Correlation plot of colonic butyrate concentration (measured in μM per gram of intestinal contents) against liver index measured in the control group pigs at days 7 and 14 PW (n = 16). The dots are coloured by the diet (control pink). Pearson’s rho is reported above the regression line. Panel C: Correlation plot of colonic butyrate concentration (μM/gram of intestinal contents) against liver index measured in the yeast group pigs at days 7 and 14 PW (n = 16). The dots are coloured by the diet (yeast dark cyan). Pearson’s rho is reported above the regression line. [file 42523_2020_31_MOESM8_ESM.docx]
